# Broadband high-frequency activity initializes distractor suppression

**DOI:** 10.1093/cercor/bhaf319

**Published:** 2025-11-26

**Authors:** Paul Schmid, Christoph Reichert, Mandy V Bartsch, Stefan Dürschmid

**Affiliations:** Leibniz Institute for Neurobiology, Brenneckestr, 6, Magdeburg 39118, Saxony Anhalt, Germany; Leibniz Institute for Neurobiology, Brenneckestr, 6, Magdeburg 39118, Saxony Anhalt, Germany; Leibniz Institute for Neurobiology, Brenneckestr, 6, Magdeburg 39118, Saxony Anhalt, Germany; Donders Center for Cognitive Neuroimaging, Donders Institute for Brain Cognition and Behaviour, Radboud University, Kapittelweg, 29, Nijmegen 6525 EN, the Netherlands; Leibniz Institute for Neurobiology, Brenneckestr, 6, Magdeburg 39118, Saxony Anhalt, Germany; Department of Psychology, University of California Berkeley 130 Barker Hall, Berkeley, CA 94720, United States; Helen Wills Neuroscience Institute, University of California Berkeley, 175 Li Ka Shing Center, MC#3370, Berkeley, CA 94720, United States

**Keywords:** broadband high-frequency activity, distractor suppression, magnetoencephalography

## Abstract

Selective attention requires fast and accurate distractor suppression. We investigated if broadband high-frequency activity (BHA; 80–150 Hz), indicative of local neuronal population dynamics in early sensory cortices, indexes rapid processing of distracting information. In a first experiment we tested whether BHA distinguishes targets from distracting information in a visual search paradigm using tilted gratings as targets and distractors. In a second experiment, we examined whether BHA distractor processing can be trained by statistical regularities. In both experiments, BHA preceded the target enhancement (N_T_) and distractor suppression (P_D_; 1–40 Hz) event-related field (ERF) components and distinguished between targets and distractors. Only the BHA but not ERF component amplitude correlated with participants’ performance and was higher for lateral distractors versus lateral targets. Furthermore, BHA predicted the strength of the P_D_. These results indicate that BHA initiates stimulus discrimination via distractor suppression.

## Introduction

Fast distractor suppression is a key aspect of selective attention alongside accurate target selection (Wöstmann et al. 2019). Searching for targets amid competing distractors increases reaction times (RT) and errors (Gutteling et al. 2022; Zhao et al. 2023) indicating spatial interference (Feldmann-Wüstefeld et al. 2021), which scales with the proximity to the target (Mounts 2000; Mathôt et al. 2010; Hickey and Theeuwes 2011). Visual search for targets is facilitated by prior knowledge about the characteristics of the targets, but also about distractors (Müller et al. 1995; Watson and Humphreys 2000; Olivers and Humphreys 2003; Arita et al. 2012; Donohue et al. 2018). Hence, success in target selection depends on fast selection and suppression of distractors (Arita et al. 2012). Selection of targets from competing distractors is addressed by a growing number of studies assessing the spatial and temporal relationships of target and distractor processing (Hickey and Theeuwes 2011; Donohue et al. 2018; Bartsch et al. 2021). In contrast to target enhancement (Luck and Hillyard 1994; Eimer 1996; Hopf et al. 2000; Woodman et al. 2009), neural mechanisms of distractor suppression are less understood.

The understanding of the neural mechanisms of attentional selection of visual stimuli is mostly based on event-related potentials/fields (ERPs/ERFs), such as the N2pc (Luck and Hillyard 1994; Eimer 1996; Hopf et al. 2000). The N2pc is an ERF, characterized by lower-frequency component (1–40 Hz) elicited at posterior electroencephalography (EEG) electrodes/magnetoencephalography (MEG) sensors between 200 and 300 ms by stimulus arrays with lateral targets and distractors in opposite hemifields. Stimulus arrays containing a lateral target while the distractor is presented on the vertical meridian and vice versa can decompose the N2pc into target enhancement (target negativity; N_T_) and distractor suppression (distractor positivity; P_D_) components (Hickey et al. 2009). Previous research (Gaspar and McDonald 2014; Gaspelin and Luck 2018; Gaspelin et al. 2023) suggests a greater amplitude for the N_T_ than P_D_ despite the necessity of fast and efficient suppression of distracting information. This raises the question of whether there might be another initial target-distractor distinction. Broadband high-frequency activity (BHA; 80–150 Hz) is a crucial analytical signal in human intracranial recordings, reflecting local neuronal population dynamics (Ray et al. 2008; Leszczyński et al. 2020; Leonard et al. 2024), with a rapid response modulation (< 200 ms) following stimulus onset (Bartoli et al. 2019; Gerber et al. 2017; Golan et al. 2017; Vishne et al. 2023), preceding the occurrence of microsaccades (Yuval-Greenberg et al. 2008). Furthermore, intracranial studies on attentional selection show a lateralized BHA to attended stimuli (Szczepanski et al. 2014) occurring before the ERPs, rendering the BHA a suitable candidate to initiate early attentional distractor suppression.

Prior knowledge about distracting information, such as distractor location, can reduce distractor interference (van Moorselaar and Slagter 2020; Richter et al. 2024). Statistical learning facilitates the establishment of an internal model that suppresses the attentional priority of locations where distractors are more likely to appear (Wang and Theeuwes 2018; Richter et al. 2024). EEG studies have demonstrated that P_D_ amplitude can be modulated by implicit learning of a frequent distractor location (van Moorselaar and Slagter 2019; van Moorselaar et al. 2020). If the BHA reflects distractor suppression, we would expect both the BHA and P_D_ to be modulated by statistical regularities.

In two experiments, we used a visual search array, containing target and distractor gratings with different orientation angles, to investigate whether the BHA serves as an early indicator of distractor suppression during target discrimination. In a second experiment, we increased the probability of distractors appearing at locations closest to the target to test whether BHA is sensitive to the spatial probability of distractor locations. Our results suggest that indeed, the BHA initiates a distractor suppression mechanism that is trainable by statistical regularities.

## Methods

### Participants

After providing their informed consent, 26 subjects participated in experiment 1, where nine were excluded from the analysis due to excessive blink and movement artifacts, resulting in a sample size of *n* = 17 (10 female, *range*: 18–38 years, *M* = 26.29 years, *SD* = 4.92 years). Thirty subjects participated in experiment 2 (21 female, *range*: 19–39, *M* = 25.47 years, *SD* = 4.03 years). Sample sizes are comparable with previous studies investigating the N2pc and its subcomponents (Hilimire et al. 2011; Gaspelin and Luck 2018; Marturano et al. 2020). All participants reported normal or corrected to normal vision and no history of neurological or psychiatric diseases. The recordings took place at the Department of Neurology, Otto-von-Guericke University Magdeburg and were approved by the local ethics committee (“Ethical Committee of the Otto-von-Guericke University Magdeburg”; approval number 34/21). All participants were compensated with 28€.

### Experimental design

#### Stimuli

**Fig. 1:**
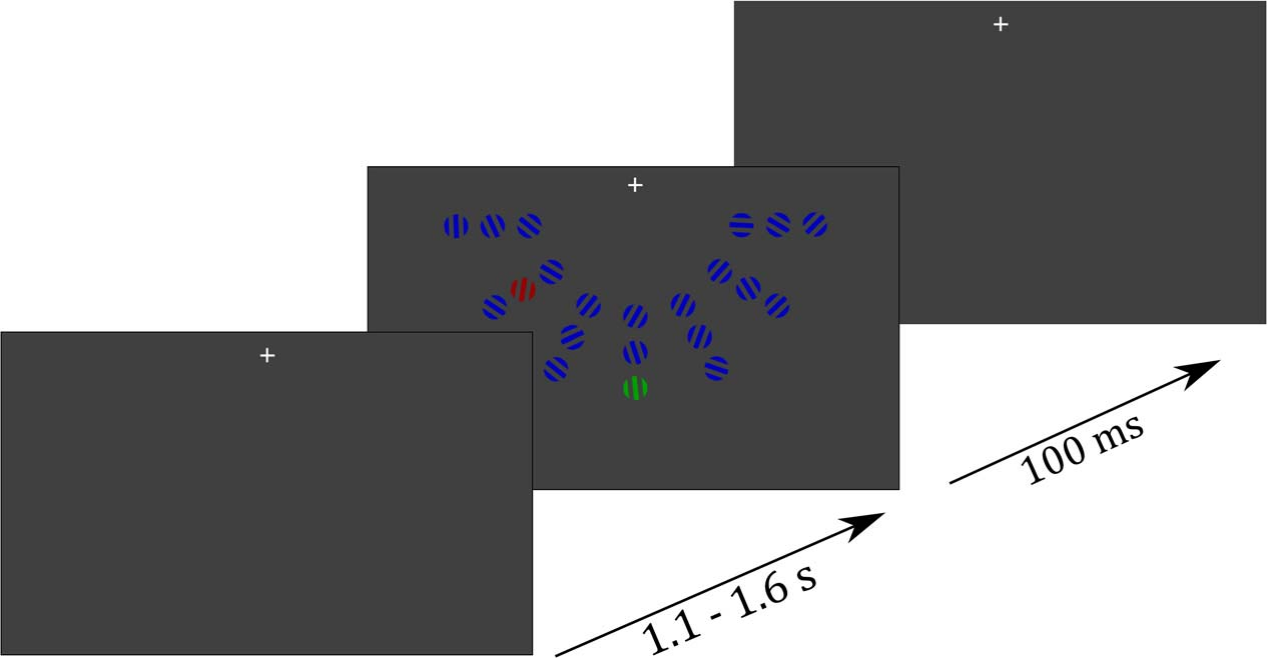
Example of a trial. Participants were presented with a fixation cross for 1.1–1.6 s, followed by presentation of a search array, containing 19 nontargets, 1 target and one pop-out distractor for 100 ms. In each block the target color was changed between green and red. In blocks with the green grating serving as the target the red grating served as the pop-out distractor and vice versa. After presentation of the search array, participants responded via button press whether the target grating was oriented to the left or right.

The participants were presented with a visual search array composed of red, green and blue grating patterns, each consisting of three stripes as viewed through a circular aperture, displayed behind a gray foreground (see Fig. 1). Red and green gratings were alternated as targets and distractors randomly between blocks, while blue gratings served as nontargets. If the red grating was the target, the green grating was the distractor and had to be ignored and vice versa. Search arrays always consisted of 19 nontargets, one pop-out target and one pop-out distractor arranged in seven columns with three gratings each and were displayed below a fixation cross to ensure a strong N2pc response (Luck et al. 1997; Hilimire et al. 2011). Participants were asked to fixate the cross, which was located at 3.75° visual angle (va) above the search array. The size of each grating was 0.54° va, and within a column they were spaced 0.1° va apart from each other. Target and distractor gratings could be tilted left or right in 10 steps of 1.5°, ranging from 1.5° to 15°, such that across all trials, there was a continuous target-distractor difference ranging from 0° to 30° in 21 steps. The angle of each individual nontarget was randomly determined, ranging from 0° to 90° (see Fig. 1). Stimulus generation and experimental control was done using MATLAB R2019 (Mathworks, Natick, USA) and the Psychophysics Toolbox (Brainard 1997). Colors were matched for isoluminance using heterochromatic flicker photometry (Lee et al. 1988).

#### Procedure

At the beginning of each of the six blocks, the participants were instructed to either attend the red and ignore the green grating or vice versa in both experiments. The task was to report via button press whether the target was oriented to the left or the right side using the right index and middle finger, respectively. Target color assignment varied in a pseudorandom order with 50% of the blocks containing red as the target color and the other half containing green as the target color. To distinguish between a lateral brain response to target and distractor, only one of them can be presented in a lateralized manner. This means if the target was presented on one of the three outer left or right columns, the distractor was presented on the vertical meridian. In the MEG, this leads to a lateralized target response. To determine the distractor response, the distractor was presented on one of the six lateralized columns while the target was presented on the vertical meridian column. Lateral target and distractor trials varied in a pseudorandom order with 50% of the trials containing lateral target and central distractor and vice versa. Eccentricity of lateral target and distractor was counterbalanced, so that the stimuli were presented equally often at the different lateral positions. Each trial started with the presentation of a fixation cross for 1350 ms (± 250 ms) before the search array was presented for 100 ms. Participants were asked to respond as fast as possible to the orientation of the target grating. Each of the six blocks contained 252 trials. In experiment 1 the positions of the lateral distractors varied randomly, while in experiment 2 half of the blocks functioned as spatial-bias blocks in a pseudorandomized block order. Previous studies have shown that the interfering distractor effect increases with the proximity between distractor and target: RT slowed, discrimination performance decreased, and P_D_ increased with proximity to the target (Hickey and Theeuwes 2011; Feldmann-Wüstefeld et al. 2021). Thus, to maximize measurable distractor suppression responses, we chose the positions closest to the target for our spatial bias condition. Specifically, in 65% of trials in spatial-bias blocks the distractor was presented on one of the six locations closest to the vertical meridian if the target was presented centrally. This analysis aimed to test whether BHA gradually adapts to the increased likelihood of a distractor location. Based on previous findings that a higher P_D_ amplitude reflects stronger distractor suppression (Gaspar and McDonald 2014; Gaspelin and Luck 2018), we similarly expected BHA—as an electrophysiological marker of distractor suppression—to show increased amplitudes in blocks with statistically biased distractor locations. If participants were able to implement stronger suppression at high-probability locations, this should be reflected in enhanced BHA signals to lateralized distractors in biased blocks compared to neutral blocks.

Note, that the moderate spatial bias (65%) was designed to introduce a “global” location bias (overall higher probability for the distractor to appear at near locations) but was unlikely to introduce strong, systematic trial-by-trial priming effects where the distractor location would have been systematically repeated on subsequent trials.

### MEG and eye movement recordings

In both experiments, participants were provided with metal-free clothing and were seated in a dimmed, magnetically shielded recording booth. Stimuli were presented on a rear-projection screen with a viewing distance of 100 cm to the participant using an LCD projector. Responses were given with the left and right hand via an MEG-compatible VPixx (VPixx Technologies, Saint-Bruno, Canada) response system. Acquisition of MEG data was performed using a whole-head Elekta Neuromag TRIUX MEG system (Elekta Oy, Helsinki, Finland), which contains 102 magnetometers and 204 planar gradiometers. Sampling rate was set to 1000 Hz. Vertical EOG was recorded bipolar with electrodes placed above and under the right eye. Horizontal EOG was recorded bipolar with electrodes placed on the left and right outer canthus. In experiment 2, we furthermore recorded participants’ eye movements in a subset of 23 participants using an Eyelink 1000 Plus system (SR Research) with a Long-Range Mount configuration and a built-in camera. We attached the Long-Range Mount configuration to the presentation screen, so that all participants were seated in the same distance to the eye tracking camera (100 cm). We recorded pupil dilation of the participants’ left eye with a sampling rate of 500 Hz. Prior to data collection, participants completed an eye tracker calibration with the built-in 9-point grid method. Preparations and recording took ~2.5 hours.

### Preprocessing

All preprocessing steps were conducted using MATLAB 2013b. Maxwell filtering was applied to reduce external noise and MEG and EOG data were down-sampled to 500 Hz. The 102 magnetometers were involved in our analyses. All filtering (see below) was performed using zero phaseshift IIR filters (fourth order; filtfilt.m in MATLAB). We filtered the EOG data between 1 and 40 Hz using a Butterworth bandpass filter. Trials with a variance that exceeded 4 times the mean variance were excluded. We also reduced artifact activity stemming from eye movements from the ongoing signal, using a linear integration approach (Parra et al. 2005). Data were epoched from 1 s preceding the stimulus presentation to 2 s after the presentation onset—sufficiently long to prevent edge effects during filtering. Each trial was then baseline-corrected relative to the 500 ms interval preceding the stimulus presentation. MEG data were bandpass filtered between 1 and 200 Hz. Then, we notchfiltered the data to discard line noise (50 Hz and three harmonics). To discard trials of excessive, nonphysiological amplitude, we used a threshold of 3pT, which the absolute MEG values must not exceed. We then visually inspected all data, excluded epochs exhibiting excessive muscle activity, as well as time intervals containing artifactual signal distortions, such as signal steps or pulses. On average, 1.6% of the trials in experiment 1 and 1.2% in experiment 2 were rejected due to artifacts.

### Statistical analysis

For statistical analysis, we conducted the following analysis steps. In Experiment 1, we analyzed behavioral performance and RT for lateralized targets, distractors, and varying target-distractor angular differences (*I*). We assessed correlations between performance and ERF N_T_ and P_D_ components (*II*), identified stimulus-related MEG-BHA channels (*III*), and compared BHA responses to lateral targets and distractors (*IV*). We then examined BHA response characteristics relative to N_T_ and P_D_ components (*V*) and explored potential lateralization of BHA responses (*VI*).

In Experiment 2, we first evaluated our implicit biased-locations manipulation influenced participants’ performance (*VII*). We extracted P_D_, N_T_, and BHA responses (*VIII*), and compared their temporal evolution with microsaccades (*IX*). Next, we tested whether P_D_ and BHA amplitudes varied with distractor location bias (*X*) and finally assessed whether BHA, N_T_ and P_D_ amplitudes were modulated by the eccentricity of lateral target and distractor positions (*XI*).

To determine statistical significance, we compared each statistical parameter against a surrogate distribution. For the grand average responses of N_T_, P_D_ and BHA, we compared the observed data against a surrogate distribution as well. In 1000 iterations, we constructed the surrogate distribution by circularly shifting time series of participants between −1 and 2 s separately. We then compared the original data with this surrogate distribution by calculating the cumulative distribution function, which resulted in a *P* value for each time point indicating whether there was a significant amplitude modulation compared to the baseline.

## Experiment 1

### I—Behavioral performance

*Distractor effect.* As a first step, we examined whether our experimental paradigm reliably elicited a distractor effect. To this end, we conducted a pilot experiment with 14 participants. The procedure was identical to the main task, except that participants completed four blocks of 252 trials each. At the beginning of each block, a cue indicated whether participants should attend to the green or the red grating. Within each block, participants reported the orientation of the target grating (left- or right-tilted) via button press. In two blocks, only the target was presented among blue non-targets (singleton condition), whereas in the remaining two blocks the target appeared together with a color distractor embedded among blue non-targets (distractor condition). Conditions were counterbalanced across subjects. Performance was assessed by comparing percentage of correctly reported target orientations and RT between the singleton and distractor conditions.

*Discrimination performance*. We compared performance (percentage of correctly reported target orientation) between lateral and central targets. First, we tested whether a greater orientation angle of the target leads to an improvement in performance. We compared performance differences for different target angles using a one-way ANOVA with the factor stimulus angle (1.5° to 15° in 10 steps of 1.5°). We then grouped performance for trials with low (≤ 7.5°) and high (≥ 9°) target angles and compared them using a *t*-test. To quantify the distractor effect, we compared whether the target discrimination performance changed with the angular difference between the orientation angles of target and distractor. To anticipate low (≤ 7.5°) and high (≥ 9°) target angles corresponds to subthreshold (< 70%; see Results) and suprathreshold performance (> 80%), respectively. Hence, in the following we use subthreshold and suprathreshold performance referring to low and high target angles, respectively. In a next step we tested the distractor effect in high-angle targets since only targets with high angles allow strongest angular differences (eg a small target angle of 1.5° to the left would allow only for a maximal angular difference of 16.5° when the distractor is tilted 15° to the right). Angular differences between target and distractor, ranging from 0° to 30°, were divided in two halves (low vs. high, see *Results*). We averaged participants’ performance for high target angles and compared them between low and high angular differences using a *t*-test.

*Reaction times.* We carried out the same analysis steps as for the performance comparisons. First, we compared RT between lateral targets and distractors using a one-way ANOVA. Post-hoc, we grouped RT for trials with subthreshold and suprathreshold performance target angles and compared them using a *t*-test. In a last step we averaged participants’ RT for suprathreshold stimuli and compared them between low and high angular differences using a *t*-test.

### I‌I—N_T_ and P_D_ response

The N_T_/P_D_ response was determined in the following way (Boehler et al. 2011): For each participant, we averaged MEG data separately for trials with targets/distractors presented in the right and left visual field. The N_T_/P_D_ was quantified in sensors showing strongest mean activity between 200 ms and 400 ms after stimulus presentation in corresponding efflux/influx zones. The signal of the sensor corresponding to the influx was subtracted from the signal of the sensor corresponding to the efflux. We then calculated the difference wave by subtracting MEG activity to targets in the right visual field form targets in the left visual field. To better compare N_T_ and P_D_ responses, we presented both P_D_ and N_T_ data as positive going responses (by inverting the sign of the N_T_ waveform). For better comparison of the response characteristics of the ERF components with the BHA, we z-standardized N_T_ and P_D_ components by performing a baseline correction to correct for both zero mean and unit standard deviation in the baseline period, ie at each time point we subtracted the baseline mean from the ERF component signal and divided the result by the standard deviation of the baseline. To assess the time range of significant N_T_/P_D_ response, we compared each time point of the observed modulation between 100 and 400 ms with a surrogate distribution (see *Statistical Analysis*). *P* values were then corrected for multiple comparisons, using the False Discovery Rate method. In a next step, we analyzed the relationship between participants’ N_T_/P_D_ amplitude and participants’ performance using Pearson correlation coefficients, resulting in a time series of correlation coefficients. Since the N2pc has been proposed to reflect a combination of N_T_ and P_D_, we aimed to compare the components’ amplitudes. Targets and distractors were physically identical and presented in equivalent locations, enabling a direct comparison between N_T_ and P_D_. Amplitude differences could be interpreted to assess strength and timing of attentional modulation. We here compared mean N_T_ and P_D_ responses using a *t*-test to reveal possible differences in the neuronal response to lateralized targets and distractors in the ERF components.

### I‌II—broad band high frequency activity

For each trial and channel, we band-pass filtered the time series in the broadband high frequency range (80–150 Hz) and obtained the analytic amplitude Af (t) of the signal by Hilbert-transforming the filtered time series. Afterwards we identified channels showing a significant amplitude modulation in the BHA following the stimulus presentation. Since we expected a BHA modulation within the first 300 ms, we z-standardized BHA amplitude values (see *Statistical Analysis II—N_T_ and P_D_ Response)* and averaged activity for each channel in the time range between 0 and 300 ms after stimulus presentation. Channels with z-scores higher than 2 were labeled as stimulus responsive. We then averaged the BHA data across the significant MEG channels and determined the time window, in which the BHA showed a significant modulation by comparing each observed time point with the surrogate distribution (see *Statistical Analysis*). After identifying the time window of significant BHA, we identified the time point when the grand average BHA response reached its peak. We defined the increasing flank of the BHA as the time from significant BHA onset to peak, while the decreasing flank was defined as the time from BHA peak to BHA offset.

### IV—BHA response to targets and distractors

In a next step we tested whether the BHA distinguishes between lateral targets and distractors. We grouped BHA responses for trials with lateral target and distractor. For both conditions (lateral target, lateral distractor), we determined the time interval of significant BHA amplitude modulation over baseline by comparing each observed time point with a surrogate distribution (see *Statistical Analysis*). Afterwards, we compared BHA responses to lateral targets and distractors using *t*-tests. Since the BHA response is known to have a fast onset and a slowly decreasing flank, we compared mean amplitudes of the increasing and decreasing BHA flank separately. To investigate the relationship between the participants’ BHA amplitude and their performance, we calculated Pearsons’s correlation coefficients, resulting in a time series of correlation coefficients.

### V—comparison between BHA response and N_T_/P_D_ response

To investigate how the BHA response characteristics relate to those of the ERF counterparts (N_T_ and P_D_), we compared the correlation between BHA amplitude and performance with the correlation between the respective ERF components amplitude and performance. We further analyzed possible latency differences between the BHA response to lateral targets and the N_T_, as well as between the BHA response to lateral distractors and the P_D_ by calculating participants’ individual time points of peak response for each component and comparing them using *t*-tests. In a final step we analyzed the direct link between participants’ mean BHA and ERF component amplitudes by calculating the Pearson’s correlation coefficient. We also tested whether BHA amplitude predicted P_D_ and N_T_ amplitude and calculated Pearson correlation coefficients between BHA amplitude at peak time point and P_D_ and N_T_ amplitude values at each time point separately. We compared these correlation coefficients with a surrogate distribution. In 1.000 iterations, we took the BHA and ERF component values of the subjects at the time of the highest observed correlation, randomly reassigned the BHA and ERF component values to the subjects and then calculated the Pearson correlation coefficient. We then calculated the 99% Confidence Interval for the correlation coefficient resulting in a critical coefficient value. Correlation coefficients exceeding this critical value were considered as showing a significant correlation between BHA peak response and ERF component amplitude. Finally, we used Williams *t*-test to determine whether the correlation coefficients of the BHA-P_D_ correlation and the BHA-N_T_ correlation differed from each other (Williams 1959).

### VI—lateralized BHA response

In a final analysis, we investigated whether lateral targets and distractors elicit a lateralized BHA response. We first analyzed whether the grand average BHA showed a lateral response. We averaged BHA response over stimulus responsive channels (see *Statistical Analysis III—Broad Band High Frequency Activity*) located in the hemispheres contralateral and ipsilateral to the presented stimulus separately, resulting in a contralateral and ipsilateral time course of BHA response. To investigate whether BHA showed a lateral response like N_T_ and P_D_, we separated trials for lateral target and distractor, resulting in separate contralateral and ipsilateral BHA responses for lateral target and distractor. We then compared mean contralateral and ipsilateral BHA responses using *t*-tests.

## Experiment 2

### VII—behavioral consequences of biased locations

*Discrimination Performance.* We compared performance (percentage of correctly reported target orientation) in trials with lateral distractors between biased-locations and neutral conditions. We compared performance for all trials and for trials where the distractor was presented at the position with the highest occurrence probability, yielding ~250 biased-location trials and 130 neutral trials per participant. We averaged participants’ performance and compared it between biased-locations and neutral conditions using a *t*-test. In an additional analysis using a *t*-test, we tested whether participants’ performance to lateral distractors differed between high-probability and low-probability locations in the neutral blocks.

*Reaction Times.* We carried out the same analysis steps as for the performance comparisons.

### VIII—stimulus response

The procedure to characterize the N_T_ and P_D_ response in experiment 2 followed the analysis steps for the N_T_ and P_D_ response in experiment 1 (see *Statistical Analysis II—N_T_ and P_D_ Response*). Analyzing the BHA response, we followed the steps to extract the BHA response in experiment 1 (see *Statistical Analysis II—Broad Band High Frequency Activity*).

### IX—temporal evolution of microsaccades

In experiment 2, we used the built-in function of the eye tracker to extract saccadic eye movements. For each participant, we first epoched the ongoing signal from 1 s preceding the stimulus presentation to 2 s after the presentation onset. This resulted in a matrix containing information on the temporal evolution and amplitude of saccadic eye movements for each trial. We then averaged the data across trials for each participant indicating how many saccadic events occurred on average at a given time point during the trials. Since we were interested in microsaccades, we only included saccades with an amplitude < 0.5° va. To analyze whether BHA, N_T_ and P_D_ could be systematically related to the timing of microsaccades, we compared the temporal evolution of microsaccades with N_T_, P_D_ and BHA response. We calculated participants’ time points of peak N_T_, P_D_ and BHA amplitude as well as the time points at which the most microsaccades occurred and compared peak microsaccade time points with peak N_T_, P_D_ and BHA time points separately using *t*-tests. Finally, we analyzed whether microsaccade rates differed between trials with lateral target and distractor by comparing mean microsaccade rates in the time range of the observed ERF components using a *t-*test.

### X—amplitude modulation with experimental condition

We then analyzed whether the P_D_ and N_T_ components and the BHA response were modulated by statistical regularities of distractor locations. We averaged P_D_, N_T_ and BHA activity for trials with the distractor (for P_D_ and BHA to lateral distractors) and target (for N_T_ and BHA to lateral targets) being presented on one of the six locations closest to the vertical meridian for biased-locations and neutral trials separately. We then analyzed possible differences between biased-locations and neutral trials in mean P_D_, N_T_ and BHA response separately using a *t-*test. Similar as in experiment 1 (see *Statistical Analysis—IV BHA Response to Targets and Distractors*), we compared mean amplitudes of the increasing BHA flank and the decreasing BHA flank separately. As we could only include a limited number of trials in the analysis of the biased-locations data due to the nature of our statistical regularities approach, we z-standardized the P_D_, N_T_ and BHA time series (see *Statistical Analysis II—N_T_ and P_D_ Response)*.

### XI—amplitude modulation with stimulus position

In an additional analysis, we investigated whether the P_D_ and N_T_ ERF components and the BHA response were modulated by the eccentricity of lateral targets and distractors. We grouped lateral stimulus positions in three columns (inner, middle, outer), depending on their distance to the vertical meridian. We averaged P_D_, N_T_ and BHA activity for trials with the distractor (for P_D_ and BHA_distractor_) and target (for N_T_ and BHA_target_) being presented on the three columns separately. We then analyzed possible P_D_ and N_T_ mean amplitude differences between the three columns using one-way ANOVAs with the factor column. For the BHA to lateral targets and distractors we analyzed possible mean amplitude differences between the three columns using 2x3 ANOVAs with the factors flank (increasing, decreasing) and column (inner, middle, outer).

## Results

### Experiment 1

#### I—behavioral performance

**Fig. 2:**
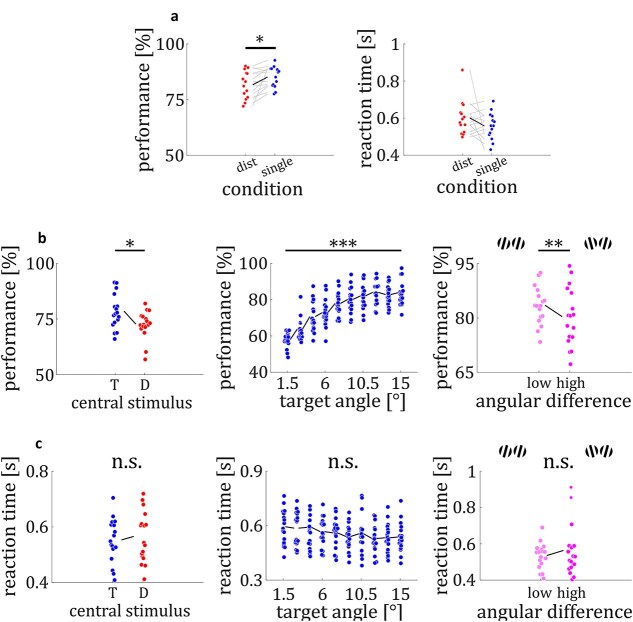
Behavioral results. a) Results pilot experiment. Participants discriminated targets better in the singleton (blue) compared to the distractor (red) condition (left). Reaction times did not differ significantly between singleton (blue) and distractor (red) condition. b) Participants discriminated central targets better than lateral targets (left). Performance increased with target orientation angle (middle). Performance for high target angle stimuli (9° to 15°) was higher for lower angular differences between target and distractor (0° to 13.5°) compared to higher angular differences (16° to 30°) (right). C: No differences in RT between trials with central target and distractor (left). No differences in RT between the different target orientation angles (middle). No differences in RT for high target angle stimuli between lower and higher angular differences (right). Colored circles represent single data points. ^*^ = *P* < 0.05. ^**^ = *P* < 0.01. ^***^ = *P* < 0.0001.

*Distractor effect.* While RT did not shorten significantly (*RT*_singleton_ = 557.4 msec; RT*_distractor_* = 601.4 msec; *t*_13_ = 1.51; *P* = 0.16; see Fig. 2a) participants discriminated targets better in the singleton condition (*M*_singleton_ = 85.1%; *M_distractor_* = 81.7%; *t*_13_ = 2.88; *P* = 0.01; see Fig. 2a) indicating that our paradigm induces a robust distractor effect.

*Discrimination performance*. We first tested whether target discrimination varied between central and lateral positions, as differences would indicate attentional modulation based on the spatial arrangement of target and distractor. Participants discriminated central targets better than lateral targets (*M*_central_ = 78.5%; *M*_lateral_ = 72.7%; *t*_16_ = 2.63; *P* = 0.02; *d* = 0.90; see Fig. 2b). A one-way ANOVA with the factor target orientation angle (1.5° to 15° in 10 steps of 1.5°) showed a significant main effect (*F*_9,160_ = 32.69; *P* < 0.0001) with higher orientation angle increasing performance (see Fig. 2b). Performance for trials with subthreshold (≤ 7.5°; see Methods) targets was significantly lower than for suprathreshold (≥ 9°) targets (*M*_sub_ = 68.7%; *M*_supra_ = 82.3%; *t*_16_ = 19.75; *P* < 0.0001; *d* = 6.77). We tested whether the orientation angle difference between target and distractor modulated discrimination performance (see Fig. 2b). High target orientation angles can have both a low and high angular difference relative to distractors. Hence, we evaluated target discrimination performance by examining angular differences for suprathreshold targets (≥ 9°; see Fig. 2b). We averaged performance for trials with low (≤ 13.5°) and high (≥ 16.5°) target-distractor angular difference, separately. We found a significant difference between low and high angular differences (*M*_low_ = 83.5%; *M*_high_ = 80.4%; *t*_16_ = 3.17; *P* = 0.006; *d* = 1.09; see Fig. 2b), with higher performance in low angular difference trials.

*Reaction times*. Reaction times did not differ between trials with lateral targets vs. lateral distractors (*M*_Target_ = 553 ms *M*_Distractor_ = 566 ms; *t*_16_ = 1.07; *P* = 0.30; *d* = 0.37; see Fig. 2c). A one-way ANOVA with the factor target orientation angle (1.5° to 15° in 10 steps of 1.5°) did not show a significant main effect (*F*_9,160_ = 1.13; *P* = 0.34). In a next step, we averaged RT for suprathreshold target trials for low and high angular differences separately. We did not find any differences in RT between low and high angular differences (*M*_low_ = 536 ms; *M*_high_ = 564 ms; *t*_16_ = 1.16; *P* = 0.26; *d* = 0.40; see Fig. 2c).

#### I‌I—N_T_ and P_D_ response

**Fig. 3:**
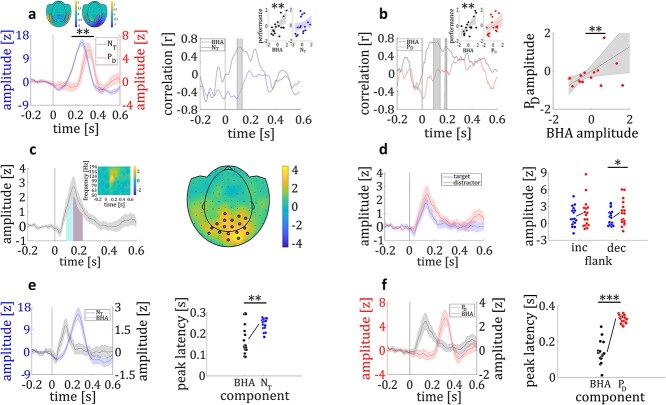
MEG results. a) Time course of the N_T_ (blue) and P_D_ (red) responses to lateral targets and distractors, respectively (left panel). Mean amplitude of the N_T_ response was higher compared to the P_D_. upper left (N_T_) and right (P_D_) inset show the topographical distribution of MEG activity to lateral targets and distractors, respectively, with corresponding efflux/influx zones. Correlation coefficient at each time point between participants’ BHA response to lateral targets (black) and their performance and between participants’ N_T_ (blue) and their performance (right panel). Insets show correlation coefficient between participants’ performance and their mean BHA and N_T_, respectively. b) Correlation coefficient at each time point between participants’ BHA response to lateral distractors (black) and their performance and between participants’ P_D_ (red) and their performance (left panel). Insets show the correlation between participants’ performance and their mean BHA and P_D_, respectively. Maximal correlation between P_D_ (at 344 ms) and peak BHA response (at 122 ms) (right panel). c) Time course of overall BHA response, showing time intervals of increasing (cyan) and decreasing (purple) flank (left panel) and its time-frequency representation (small inset). Topographical distribution of overall BHA response with sensors showing a significant response being highlighted (right panel). d) Time course of BHA response to lateral targets (blue) and distractors (red) (left panel). Individual mean BHA amplitude for lateral targets and distractors for the increasing and decreasing BHA flank (right panel). BHA amplitude of the decreasing flank was higher for lateral distractors compared to targets. e) Time course for BHA to lateral targets (black) and N_T_ (blue) (left panel). Individual time points of peak BHA and N_T_ response (right panel). f) Time course for BHA to lateral distractors and P_D_ (third panel). Individual time points of peak BHA and P_D_ response (right panel). Colored circles represent single data points. Shaded colored lines represent the standard errors of the means (SEM). ^*^ = *P* < 0.05. ^**^ = *P* < 0.01. ^***^ = *P* < 0.001.

We identified time windows of significant N_T_ and P_D_ responses and investigated whether they varied with behavioral performance. Lateral targets elicited a N_T_ response between 166 and 330 ms (z_crit_ = 3.47; N_Tmax_ = 15.24 at 258 ms; all *P* < 0.04; see Fig. 3a). The N_T_ amplitude was not correlated with participants’ performance, neither for the time resolved analysis (*r*_max_ = 0.40 at 180 ms; all *P* > 0.11; see Fig. 3a), nor for correlation with N_T_ averaged across the time interval of significant N_T_ response (*r* = 0.24; *P* = 0.36; see Fig. 3a).

Lateral distractors elicited a P_D_ between 260 and 370 ms (z_crit_ = 1.43; P_Dmax_ = 5.43 at 320 ms; all *P* < 0.02; see Fig. 3a). The P_D_ amplitude was not correlated with participants’ performance either, neither in a time-resolved manner (*r*_max_ = 0.34 at 324 ms; all *P* > 0.19; see Fig. 3b), nor for averaged P_D_ response (*r* = 0.37; *P* = 0.14; see Fig. 3b). We found the amplitude of the N_T_ twice as large as the P_D_ (*M*_NT_ = 10.80; *M*_PD_ = 4.22; *t*_16_ = 3.55; *P* = 0.003; see Fig. 3a). Furthermore, the N_T_ response (~246 ms; *SD* = 27 ms) peaked significantly earlier compared to the P_D_ response (~328 ms; *SD* = 23 ms; *t*_16_ = 10.44; *P* < 0.0001; *d* = 3.58).

#### I‌II—broad band high frequency activity

A total of 18 occipital magnetometers showed a significant BHA response to lateral stimuli between 84 and 204 ms (z_crit_ = 0.85; BHA_max_ = 2.73 at 136 ms; all *P* values < 0.05; see Fig. 3c).

#### IV—BHA response to targets and distractors

We found a significant BHA response to lateral targets between 96 ms and 180 ms (z_crit_ = 0.57; BHA_max_ = 1.76 at 134 ms; all *P* < 0.05; see Fig. 3d) and a significant BHA response to lateral distractors between 92 and 206 ms (z_crit_ = 0.99; BHA_max_ = 2.43 at 142 ms; all *P* < 0.04; see Fig. 3d). The increasing flank (84 ms—134 ms, see Fig. 3c) of the BHA response did not show differences between lateral targets and distractors (*M*_Target_ = 1.27; *M*_Distractor_ = 1.84; *t*_16_ = 1.73; *P* = 0.10; *d* = 0.59). However, BHA amplitude of the decreasing flank (134 ms—204 ms) was higher for lateral distractors compared to lateral targets (*M*_Target_ = 1.10; *M*_Distractor_ = 1.92; *t*_16_ = 2.31; *P* = 0.035; *d* = 0.90; see Fig. 3d).

#### V—comparison between BHA response and N_T_/P_D_ response

In a following step, we compared the response characteristics of the ERF target enhancement and distractor suppression components with the BHA’s response characteristics.

*BHA response to lateralized targets* vs. *N_T_.* In contrast to the N_T_ amplitude, the BHA amplitude to lateral targets was correlated to individual performance both for the time-resolved analysis (*r*_max_ = 0.67 at 118 ms; all *P* < 0.0125; see Fig. 3a) and the averaged BHA to targets (*r* = 0.65; *P* = 0.005; see Fig. 3a). In the next step, we tested whether the relationship between BHA and performance remains when controlling for N_T_ (*r* = 0.65; *P* = 0.007) or P_D_ (*r* = 0.61; *P* = 0.01), and whether N_T_ (*r* = 0.29; *P* = 0.28) or P_D_ (*r* = 0.004; *P* = 0.99) predicts performance when controlling for BHA. The BHA (~174 ms; *SD* = 70 ms) peaked earlier than the N_T_ (~246 ms; *SD* = 27 ms; *t*_16_ = 3.72; *P* = 0.002; *d* = 1.28; see Fig. 3e). Neither mean, nor peak BHA response was correlated to mean (*r* = −.08; *P* = 0.76) and peak (*r*_max_ = 0.33 at 168 ms; *P* = 0.19) N_T_ response, respectively.

*BHA response to lateralized distractors* vs. *P_D_*. The BHA to lateral distractors (at ~ 142 ms; *SD* = 64 ms) peaked earlier than the P_D_ (~328 ms; *SD* = 23 ms; *t*_16_ = 7.51; *P* < 0.0001; *d* = 2.58; see Fig. 3f). Both mean and peak BHA response were correlated to mean (*r* = 0.57; *P* = 0.017) and peak (*r*_max_ = 0.64 at 344 ms; *P* = 0.006; see Fig. 3b) P_D_ response, respectively. The BHA amplitude to lateralized distractors, but not the P_D_, was correlated to performance (time-resolved analysis: *r*_max_ = 0.74 at 122 ms; all *P* < 0.0125; average BHA amplitude: *r* = 0.70; *P* = 0.002; see Fig. 3b). Running a partial correlation analysis between BHA and behavioral performance, controlling for P_D_ amplitude, we found a significant relationship between BHA and performance (*r* = 0.61; *P* = 0.01). Running the correlation analysis between P_D_ and performance while controlling for BHA amplitude revealed no significant correlation between P_D_ and performance (*r* = 0.004; *P* = 0.99). Williams *t*-test revealed a significant difference between the correlation coefficients of the BHA-N_T_ (*r* = −.08) and the BHA-P_D_ (*r* = 0.57) correlation (*t*_14_ = 2.16; *P* = 0.049; *d* = 0.74), indicating that the BHA is significantly stronger correlated to the P_D_ component compared to the N_T_ component.

#### VI—lateralized BHA response

**Fig. 4:**
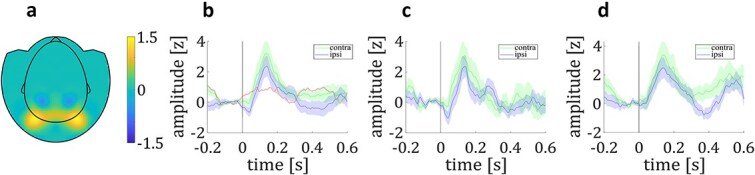
Lateralized BHA response. a) Topographical distribution of overall BHA response (contralateral minus ipsilateral). b) Contralateral and ipsilateral BHA response to lateral stimuli (target and distractor). Red line shows the difference wave (contralateral minus ipsilateral). c) Contralateral and ipsilateral BHA response to lateral targets. d) Contralateral and ipsilateral BHA response to lateral distractors. Shaded colored lines represent the standard error of the means (SEM).

Seven stimulus-responsive channels were located in the left hemisphere and nine in the right hemisphere (see *Results III—Broadband High Frequency Activity*; see Fig. 4a). When we compared mean contralateral and ipsilateral grand average BHA responses in the time range of the decreasing flank, we found a higher BHA response contralateral to the presented stimulus compared to ipsilateral (*M*_contra_ = 2.47; *M*_ipsi_ = 1.64; *t*_16_ = 2.22; *P* = 0.04; *d* = 0.76; see Fig. 4b). BHA response to lateral targets did not significantly differ between contralateral and ipsilateral hemisphere (*t*_16_ = 1.24; *P* = 0.23; *d* = 0.43; see Fig. 4c), neither did BHA response to lateral distractors (*t*_16_ = 0.61; *P* = 0.55; *d* = 0.21; see Fig. 4d).

In sum, we found participants’ behavioral performance to increase with increasing target angle and higher performance for trials with lower angular difference between target and distractor grating. We further found that the BHA response differed between lateral targets and distractors and was correlated to participants’ individual performance and P_D_ amplitude.

### Experiment 2

#### VII—behavioral consequences of biased distractor location

**Fig. 5:**
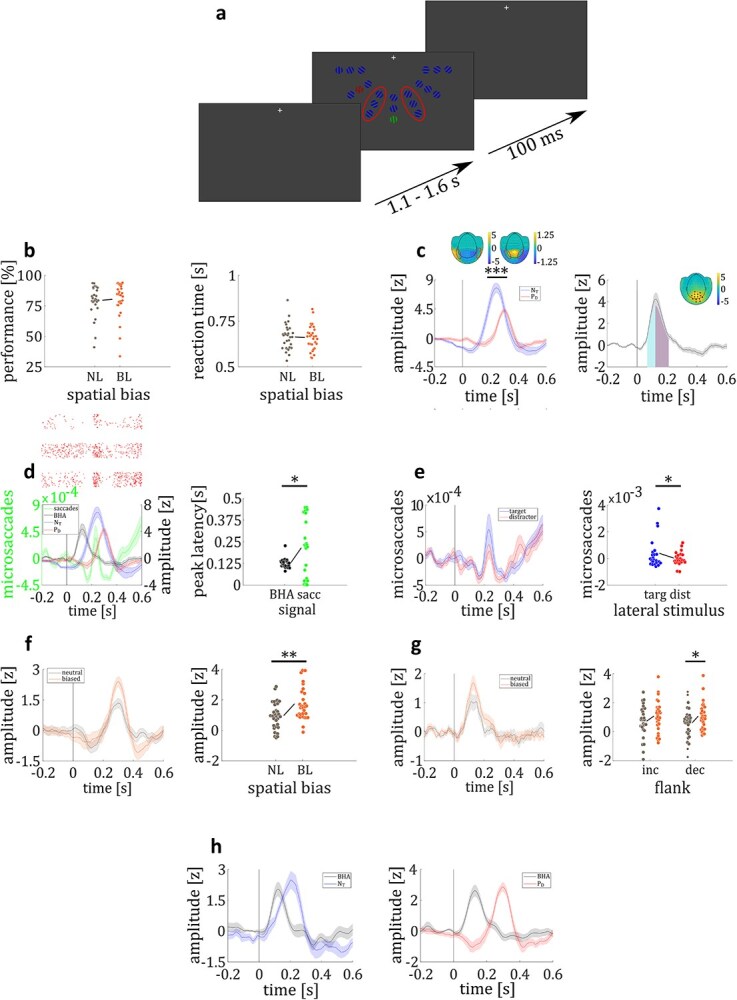
Statistical regularities experiment. a) Depiction of the paradigm. Red ellipses (not visible in experiment) mark distractor positions closest to the midline used for biased locations. b) Performance (left panel) and RT (right panel) did not differ between biased-locations and neutral trials. c) Time course of the N_T_ (blue) and P_D_ (red) grand average responses to lateral targets and distractors, respectively (left panel). The N_T_ response was higher compared to the P_D_ response. Upper left (N_T_) and right (P_D_) inset show the topographical distribution of MEG activity to lateral targets and distractors, respectively, with corresponding efflux/influx zones. Time course of overall BHA response, showing time intervals of increasing (cyan) and decreasing (purple) flank (right panel). Topographical distribution of overall BHA response with sensors showing a significant response being highlighted (small inset). d) Time course of microsaccadic eye movements (< 0.5° va), BHA, N_T_ and P_D_ response (left panel). Red dots mark single microsaccadic events of three example participants (upper inset, three rows). Individual time points of peak BHA and microsaccade response (right panel). e) Time course of microsaccades to lateral targets and distractors (left panel). Individual mean microsaccade rate to lateral targets and distractors (right panel). f) Time course of P_D_ response in biased-locations and neutral condition (left panel). Individual mean P_D_ amplitude for biased-locations and neutral trials, for distractors close to the midline. Mean P_D_ response was higher in biased-locations compared to neutral trials (right panel). g) Time course of BHA response in biased-locations and neutral condition (left panel). Individual mean BHA amplitude for biased-locations and neutral trials for the increasing and decreasing BHA flank (right panel). BHA amplitude of the decreasing flank was higher for biased trials compared to neutral trials. h) Time course of N_T_ (blue) and BHA (black) response to lateral targets presented on the inner column (left panel). Time course of P_D_ (red) and BHA (black) response to lateral distractors presented on the inner column (right panel). Colored circles represent single data points. Shaded colored lines represent the standard error of the means (SEM). ^*^ = *P* < 0.05. ^**^ = *P* < 0.01. ^***^ = *P* < 0.0001.

We first examined whether our implicit biased-locations manipulation (see Fig. 5a) had an effect on participants’ behavioral measures of performance.

*Discrimination performance*. Performance did not differ between biased-locations and neutral conditions, neither for all lateral distractor trials (*M*_biased_ = 80.5%; *M_neutral_* = 80.2%; *t_29_* = 0.19; *P* = 0.85; *d* = 0.07), nor for trials where the distractor was presented at the position with the highest occurrence probability (*M*_biased_ = 80.4%; *M_neutral_* = 79.6%; *t_29_* = 0.53; *P* = 0.60; *d* = 0.18; see Fig. 5b). When testing whether participants’ performance to lateral distractors differed between high-probability and low-probability locations in the neutral blocks, we did not find significant differences, (*M*_high_ = 79.6%, *M*_low_ = 80.2%; *t*_29_ = 1.08; *P* = 0.29).

*Reaction times*. Reaction times did not differ between biased-locations and neutral conditions, neither for all lateral distractor trials (*M*_biased_ = 659 ms; *M_neutral_* = 661 ms; *t_29_* = 0.55; *P* = 0.59; *d* = 0.19), nor for trials where the distractor was presented at the position with the highest occurrence probability (*M*_biased_ = 660 ms; *M_neutral_* = 663 ms; *t_29_* = 0.69; *P* = 0.50; *d* = 0.24; see Fig. 5b). Reaction times to lateral distractors did not differ between high-probability and low-probability locations in the neutral blocks (*M*_high_ = 663 ms, *M*_low_ = 661 ms; *t*_29_ = 1.22; *P* = 0.23).

#### VIII—stimulus response

In experiment 2, lateral targets elicited a N_T_ response between 154 and 328 ms (z_crit_ = 1.17; N_Tmax_ = 7.74 at 244 ms; all *P* < 0.05; see Fig. 5c), while lateral distractors elicited a P_D_ response between 234 and 362 ms (z_crit_ = 0.59; P_Dmax_ = 4.29 at 298 ms; all *P* < 0.003; see Fig. 5c). Furthermore, the N_T_ showed a stronger modulation than the P_D_ response (*M*_NT_ = 5.44; *M*_PD_ = 2.97; *t*_29_ = 4.63; *P* = 0.00007; *d* = 1.59; see Fig. 5c).

A total of 14 occipital magnetometers (five in the left, seven in the right hemisphere) showed a significant BHA response to lateral stimuli between 68 and 210 ms (z_crit_ = 0.60; BHA_max_ = 4.22 at 122 ms; all *P* values < 0.05; see Fig. 5c).

#### IX—temporal evolution of microsaccades

To analyze whether BHA, N_T_ and P_D_ could be systematically related to the timing of microsaccades, we compared the temporal evolution of microsaccades with BHA, N_T_ and P_D_ response.

We compared microsaccade, BHA and N_T_ and P_D_ peak times. While we found no difference in peak times between microsaccades and N_T_ (microsaccades_peak_ = 215 ms; NT_peak_ = 251 ms; *t*_22_ = 0.98; *P* = 0.34; *d* = 0.34), we found that the P_D_ peaked later than microsaccades (PD_peak_ = 304 ms; *t*_22_ = 2.39; *P* = 0.026; *d* = 0.82). In contrast we found that BHA precedes microsaccades (microsaccades_peak_ = 215 ms; BHA_peak_ = 131 ms; *t*_22_ = 2.40; *P* = 0.025; *d* = 0.82; see Fig. 5d).

We found more microsaccades to lateral targets compared to distractors (*M*_Target_ = 0.0004; *M*_Distractor_ = 0.00003; *t*_22_ = 2.20; *P* = 0.038; *d* = 0.76; see Fig. 5e) indicating that differences in amplitude modulation of N_T_ (*M* = 5.44) and P_D_ (*M* = 2.97) might at least in part be explained by differences in microsaccade rate.

#### X—amplitude modulation with experimental condition

To analyze whether brain responses of target selection and distractor suppression could be modulated by statistical regulations of distractor locations, we compared mean P_D_ responses between biased-locations and neutral trials for distractors located close to the midline (marked positions in Fig. 5a). We found a higher P_D_ response in biased-locations trials compared to neutral trials (*M*_biased_ = 1.74; *M*_neutral_ = 0.97; *t*_29_ = 2.88; *P* = 0.007; *d* = 1.00; see Fig. 5f). The target selection, as indexed by the N_T_, showed no difference between biased-locations and neutral trials (*M*_biased_ = 2.17; *M*_neutral_ = 2.09; *t*_29_ = 0.30; *P* = 0.77; *d* = 0.72), which was expected since the statistical probability of the target locations was identical for biased-locations and neutral blocks.

While the increasing flank of the BHA (68–122 ms) showed no significant difference between biased-locations and neutral trials (*M*_biased_ = 1.08; *M*_neutral_ = 0.73; *t*_29_ = 1.44; *P* = 0.16; *d* = 0.49), we found the decreasing flank of the BHA (122–210 ms) in biased-locations trials to be significantly increased in amplitude compared to neutral trials (*M*_biased_ = 1.07; *M*_neutral_ = 0.61; *t*_29_ = 2.06; *P* = 0.0485; *d* = 0.71; see Fig. 5g).

#### SuXI—amplitude modulation with stimulus position

The following analyses were run to investigate whether N_T_, P_D_ and BHA varied with eccentricity of lateral pop-out stimuli. While mean N_T_ response did not differ between the three lateral columns (*F*_2,87_ = 0.04; *P* = 0.96), P_D_ amplitude increased with proximity between the lateral distractor and central target (*F*_2,87_ = 3.91; *P* = 0.02).

Similar to the N_T_ component, the BHA to lateral targets was not modulated by lateral target position (main effect column: *F*_2,174_ = 1.43; *P* = 0.24). A 2x3 ANOVA analyzing the increasing and decreasing flank of the BHA to lateral distractors between the three lateral distractor columns revealed a marginally significant main effect of column (*F*_2,174_ = 2.71; *P* = 0.07), indicating a trend of increasing BHA with proximity between the lateral distractor and central target.

While we found no behavioral consequences of statistical regularities of distractor locations on participants’ performance, we found the P_D_ and the BHA to lateral distractors to be increased during blocks of biased locations. Amplitude of P_D_ and BHA to lateral distractors increased with closer proximity to the central target. Analyzing eye movement data, we found that microsaccades (< 0.5°) occurred after the BHA, but in the time range of N_T_ and P_D_ components. Additionally, the rate of microsaccades increased in trials with a lateral target compared to lateral distractor.

## Discussion

We examined whether broadband high-frequency activity (BHA) is an early marker of spatial selective attention and distractor suppression. Specifically, we compared the BHA response in the MEG to N_T_ and P_D_ ERF components. We found that the BHA recorded at MEG sensors covering the occipital cortex distinguished between lateral targets and distractors with a higher amplitude to lateral distractors. The BHA preceded the ERF components by more than 100 ms. Most importantly, the early BHA modulation was correlated to individual performance and P_D_ (but not N_T_) response. In a second experiment, we examined how the P_D_ and BHA responses are affected by statistical regularities of distractor locations and compared the time course of microsaccades to BHA, N_T_ and P_D_. Both, the P_D_ and BHA to lateral distractors, increased in amplitude during biased-locations blocks indicative of a trainable distractor suppression mechanism. Microsaccades peaked later than the BHA, but in the time range of the ERF components. These results indicate that the early visual BHA, as a selection-for-rejection mechanism, reflects the initial instance of effective distractor suppression.

In general, in line with previous literature (Wienke et al. 2021), we found better target discrimination with higher target orientation angle. Participants showed faster RT and lower error rates to central compared to lateral targets indicating a location-based effect. This location-based effect is in line with a previous study in which search displays consisted of 16 letters comprising targets (colored horizontally/upright “T”), distractors (colored horizontally/upright “L”) and non-targets (gray horizontally “T”) (Hilimire et al. 2011). Since target and distractor similarity were not systematically varied, their paradigm could not identify a feature-based distractor effect. In our study, performance decreased with greater orientation difference between target and distractor angle. Reaction times did not increase, ruling out the interpretation of response interference (Maniscalco et al. 2012). While the observed increase in accuracy for trials with smaller angular differences between target and distractor may resemble a “redundant target” effect, we do not find corresponding speeded RT, which are characteristic of that phenomenon (Gondan et al. 2004; Miller et al. 2014). In fact, most participants exhibit numerically longer RT in trials with smaller angular differences. These results indicate a feature-based distractor effect based on the angular difference between targets and distractors.

Both the N_T_ and P_D_ were elicited by lateral targets and distractors, respectively. We observed an N_T_ onset around 200 ms and a peak response around 260 ms after stimulus presentation, which is in line with previous findings (Hickey et al. 2009; Donohue et al. 2018; Drisdelle and Eimer 2021). Critically, the P_D_ in our experiments peaked later than the N_T_ (~80 ms), but showed the same topography as found in Donohue et al. (2018). The P_D_ resembled an inverted N2pc field with an efflux component over central occipital areas and an influx component at lateral occipital areas. Despite the differences in stimuli in previous studies, we found a similar relationship between N_T_ and P_D_ magnitude. Donohue et al. (2018) used a symmetric search array with T letters around a fixation cross, restricting the color-defined target or distractor to lateral positions. In line with Donohue et al. (2018), we found that the neural response to targets (N_T_) is stronger than the response to distractors (P_D_). Given that distractor suppression is an essential process, one might expect its neural correlate to be comparable with the target selection response. Previous work also suggests that the microsaccade rate modulates the EEG/MEG amplitude (Yuval-Greenberg et al. 2008). Lateral targets require a spatial shift of attention for successful discrimination, which explains the higher microsaccades rate (Rucci et al. 2007). The spikes elicited by the microsaccadic movements could then add to the MEG amplitude (Lowet et al. 2018; Liu et al. 2023). Microsaccades in our study peaked around 200 ms (Yuval-Greenberg et al. 2008). In this time range microsaccades overlap with ERF components and explain their field distribution (Carl et al. 2012; Liu et al. 2023). Given that microsaccade rate to lateral targets is higher than to lateral distractors, the higher amplitude of the N_T_ might partially be explained by microsaccades. These results further indicate that specifically lower-frequency responses might be influenced by microsaccadic eye movements. The late and reduced P_D_ in addition to fewer microsaccades compared to target trials indicate that the suppression process has already been initiated earlier. To rule out oculomotor confounds, we examined the temporal relationship between BHA and microsaccades. BHA increases consistently preceded microsaccade onset, indicating that the observed neural effects were not driven by eye movements. While microsaccades can modulate EEG/MEG signals (Yuval-Greenberg et al. 2008), they are correlates—not causes—of attentional modulations (Yu et al. 2022). These findings support the interpretation that BHA reflects genuine neural processes related to attentional selection.

The BHA showed a stronger modulation to lateral distractors compared to targets. The peak BHA predicted the strength of individual P_D_ (but not N_T_) responses, which is in line with a cortical “selection for rejection” mechanism (Donohue et al. 2018; Bartsch et al. 2021). In Donohue et al., the authors proposed the N1pc as an early marker for selection or rejection. The N1pc peaked around the decreasing flank of the BHA in our study. However, in contrast to the BHA, the N1pc did not show differences in amplitude to lateral targets versus distractors. Hence, the N1pc can be seen as a general “attend-to-me” signal to salient items (Sawaki and Luck 2010). Since the BHA was only correlated to the P_D_, but not the N_T_ our results suggest that the BHA represents a more selective “selection for rejection” mechanism and thus serves as an early marker of distractor suppression.

An alternative account is that in our paradigm, no initial selection of the distractor occurred at all. In fact, the distractor color was predictable at all times which would theoretically allow for a proactive suppression. However, our behavioral pre-test confirmed that the presence of the distractor induced measurable accuracy impairments, rendering an effective pro-active suppression unlikely. In line with this, in our main experiment, performance should have been consistent across the unpredictable distractor orientations under proactive suppression. Instead, performance varied with distractor orientation and differed between central and lateral distractor locations. Furthermore, we observed a late P_D_ component, emerging 300 ms after stimulus onset and following the onset of the N_T_. This aligns with previous findings linking the late P_D_ to reactive suppression that rather occurs to terminate attentional selection of the distractor (Gaspelin et al. 2023). in addition, the neural evidence argues against an early filtering. BHA correlated significantly with behavioral performance, indicating that distractors were neurally processed and influenced behavior. Such a relationship would not be expected if anticipatory suppression had prevented distractor encoding. Together, these findings support a reactive suppression account, suggesting that distractors were at least partially processed before suppression was engaged. Indeed, since in our paradigm color did not only define the distractor but also the target, a pro-active dimension-based suppression account—as in additional singleton design, where targets and distractors differ by dimension (eg shape vs. color)- would have not been feasible. In addition, in our paradigm, the color assignment of target and distractor alternates across block, increasing the overall salience of the distractor feature. Prior findings by Donohue et al. (2018) using a similar design support reactive suppression: distractors are first selected and only later suppressed, as reflected by a late P_D_. Our data add to the pro-active versus reactive distractor suppression discussion and support previous observations, suggesting that it is not possible to pro-actively suppress a distractor when it is defined in the same feature dimension as the target but that it will instead be selected to some degree (Liesefeld et al. 2017; Donohue et al. 2018; vab Moorselaar and Slagter 2019). Last but not least, even if BHA does not directly index selection per se, its selective correlation with distractor processing aligns with multiple theoretical accounts, including attentional capture, cognitive control, task interference, or feature-specific encoding—all of which require stimulus-level processing. Together, these findings argue against proactive suppression and support a model in which salient distractors are initially selected and only subsequently suppressed.

While we found no differences in N_T_ amplitude between different lateral target positions, our findings of an increasing P_D_ with increasing proximity between target and distractor is in line with previous findings (Hickey and Theeuwes 2011; Feldmann-Wüstefeld et al. 2021). In a modified additional singleton task Feldmann-Wüstefeld et al. (2021) presented participants with a visual search display containing circular (nontargets and salient distractor) and diamond shapes (target) requiring participants to respond to the orientation of a line inside the target stimulus. Performance increased and P_D_ amplitude decreased with distance between target and distractor (Feldmann-Wüstefeld et al. 2021). The BHA to lateral distractors in our study shows a trend towards the same pattern with the BHA amplitude numerically increasing with proximity between the central target and lateral distractor. These results further support the conclusion that the BHA is involved in distractor selection for suppression.

BHA has been the focus of studies using human intracranial recordings (Miller et al. 2014) but is only recently described in non-invasive recordings (Wienke et al. 2021). Intracranial studies demonstrated how BHA varies in different visual areas (Gerber et al. 2017; Golan et al. 2017; Bartoli et al. 2019; Vishne et al. 2023). Our observed BHA response with a fast onset prior to 100 ms, a peak prior to 200 ms and a slowly decreasing flank closely resemble the BHA observed in these studies (Gerber et al. 2017; Golan et al. 2017; Bartoli et al. 2019; Vishne et al. 2023), especially the BHA response in V1 electrodes (Golan et al. 2017; Bartoli et al. 2019).

Previous intracranial recordings in epilepsy patients found the BHA to be increased during attentional selection of auditory stimuli (Ray et al. 2008). In the visual domain, previous studies investigating attentional modulation of neural activity primarily focused on broad gamma-bands between 30 and 130 Hz with mixed results (Tallon-Baudry et al. 2005; Davidesco et al. 2013). Tallon-Baudry et al. (2005) found an increase in baseline gamma-range activity (30–130 Hz) and a decrease during stimulus presentation in lateral occipital cortex, but an increase over fusiform gyrus during attentive states. In contrast, Davidesco et al. (2013) presented participants with small and large objects and cued them to attend one of the stimuli. They found activity in the gamma-range (30–90 Hz) in visual cortex to be increased during attentive states, while previous studies using a narrower gamma-range (30–50 Hz) failed to show such modulations (Chalk et al. 2010). Furthermore, Davidesco et al. (2013) found attentional modulation of gamma activity to occur earlier in early visual cortical areas compared to higher visual areas. High-frequency modulation (> 80 Hz) during selective attention was also found in intracranial recordings (Szczepanski et al. 2014). Participants were instructed to covertly attend either to the left or right visual field and respond to a target. Szczepanski et al. (2014) found an increase in BHA during attentive states over visual areas. Most interestingly, ~20% of the recorded electrodes exhibited stronger BHA responses contralateral to the attended visual field, which is in line with our results of an increased BHA in MEG sensors contralateral to lateral stimuli.

A central finding of our study is that BHA is modulated by statistical regularities of distractor locations, a relationship not yet demonstrated. In general, spatial proximity between targets and distractors increases interference (Mounts 2000; Mathôt et al. 2010; Hickey and Theeuwes 2011). Therefore, we increased the occurrence probability of distractors at positions close to the central targets to test whether the BHA/P_D_ responses could be modified by statistical regularities. Research has shown that while individuals can detect and learn statistical regularities in their environment, this learning does not always translate into enhanced performance (Fiser and Aslin 2001; Conway and Christiansen 2006; Siegelman et al. 2018). In line with these results, participants in our study showed no performance improvement. We attribute this to the target orientation angles being too small to discriminate despite a further improvement of distractor suppression. Our results indicate that the BHA to lateral distractors and the later P_D_ are enhanced by statistical regularities. In a previous study, van Moorselaar and Slagter (2019) found the P_D_ to be reduced during statistical learning. There could be several reasons for these conflicting results. First, in their study the distractor was repeatedly presented at one specific location, while the lateral distractor in our biased-locations blocks was more often presented at the six lateral locations closest to the vertical target. Thus, in our study the distractor still induced a certain level of interference since its precise location was not fully predictable. Second, repeatedly presenting the distractor at one specific location can lead to a repetition suppression effect, where lower-frequency (Grill-Spector et al. 2006) and high-frequency neural responses (Eckert et al. 2022) decrease with repetition. We can rule out this possibility since the BHA was increased in the biased-locations condition. Furthermore, our observed differences between biased-locations and neutral conditions cannot be explained by a closer proximity between target and distractor in the biased-locations condition. We conclude that the BHA was sensitive to global statistical regularities as introduced by a spatial bias in our second experiment. Due to our spatial bias paradigm in our second experiment, these results were likely not a result of inter-trial priming. Overall, our findings suggest that BHA is a selection-for-rejection mechanism that can be reinforced by statistical regularities.

## Conclusion

Our findings extend prior work on the human BHA and spatial attention. The MEG-BHA in our study showed response characteristics strongly similar to that observed in non-human primate and human intracranial recordings. The BHA distinguished between lateralized targets and distractors and preceded the lower-frequency ERF components by more than 100 ms. Furthermore, the BHA predicted participants’ performance and P_D_ response. These results indicate that the BHA is best suited to test the temporal evolution of stimulus discrimination as a key marker of successful subsequent distractor suppression.

## References

[ref1] Arita JT, Carlisle NB, Woodman GF. 2012. Templates for rejection: configuring attention to ignore task-irrelevant features. J Exp Psychol Hum Percept Perform. 38:580–584. 10.1037/a0027885. 22468723 PMC3817824

[ref2] Bartoli E et al. 2019. Functionally distinct gamma range activity revealed by stimulus tuning in human visual cortex. Curr Biol CB. 29:3345–3358.e7. 10.1016/j.cub.2019.08.004. 31588003 PMC6810857

[ref3] Bartsch MV, Merkel C, Schoenfeld MA, Hopf J-M. 2021. Attention expedites target selection by prioritizing the neural processing of distractor features. Commun Biol. 4:1–15. 10.1038/s42003-021-02305-9. 34188169 PMC8242025

[ref4] Boehler CN, Tsotsos JK, Schoenfeld MA, Heinze H-J, Hopf J-M. 2011. Neural mechanisms of surround attenuation and distractor competition in visual search. J Neurosci. 31:5213–5224. 10.1523/JNEUROSCI.6406-10.2011. 21471356 PMC6622702

[ref5] Brainard DH . 1997. The psychophysics toolbox. Spat Vis. 10:433–436. 10.1163/156856897X00357. 9176952

[ref6] Carl C, Açık A, König P, Engel AK, Hipp JF. 2012. The saccadic spike artifact in MEG. NeuroImage. 59:1657–1667. 10.1016/j.neuroimage.2011.09.020. 21963912

[ref7] Chalk M et al. 2010. Attention reduces stimulus-driven gamma frequency oscillations and spike field coherence in V1. Neuron. 66:114–125. 10.1016/j.neuron.2010.03.013. 20399733 PMC2923752

[ref8] Conway CM, Christiansen MH. 2006. Statistical learning within and between modalities: pitting abstract against stimulus-specific representations. Psychol Sci. 17:905–912. 10.1111/j.1467-9280.2006.01801.x. 17100792

[ref9] Davidesco I et al. 2013. Spatial and object-based attention modulates broadband high-frequency responses across the human visual cortical hierarchy. J Neurosci. 33:1228–1240. 10.1523/JNEUROSCI.3181-12.2013. 23325259 PMC6704891

[ref10] Donohue SE, Bartsch MV, Heinze H-J, Schoenfeld MA, Hopf J-M. 2018. Cortical mechanisms of prioritizing selection for rejection in visual search. J Neurosci. 38:4738–4748. 10.1523/JNEUROSCI.2407-17.2018. 29691330 PMC6596014

[ref11] Drisdelle BL, Eimer M. 2021. P _D_ components and distractor inhibition in visual search: new evidence for the signal suppression hypothesis. Psychophysiology. 58:e13878. 10.1111/psyp.13878. 34110022

[ref12] Eckert D et al. 2022. Distinct interacting cortical networks for stimulus-response and repetition-suppression. Commun Biol. 5:1–10. 10.1038/s42003-022-03861-4. 36064744 PMC9445181

[ref13] Eimer M . 1996. The N2pc component as an indicator of attentional selectivity. Electroencephalogr Clin Neurophysiol. 99:225–234. 10.1016/0013-4694(96)95711-9. 8862112

[ref14] Feldmann-Wüstefeld T, Weinberger M, Awh E. 2021. Spatially guided distractor suppression during visual search. J Neurosci. 41:3180–3191. 10.1523/JNEUROSCI.2418-20.2021. 33653697 PMC8026355

[ref15] Fiser J, Aslin RN. 2001. Unsupervised statistical learning of higher-order spatial structures from visual scenes. Psychol Sci. 12:499–504. 10.1111/1467-9280.00392. 11760138

[ref16] Gaspar JM, McDonald JJ. 2014. Suppression of salient objects prevents distraction in visual search. J Neurosci. 34:5658–5666. 10.1523/JNEUROSCI.4161-13.2014. 24741056 PMC6608232

[ref17] Gaspelin N, Luck SJ. 2018. Distinguishing among potential mechanisms of singleton suppression. J Exp Psychol Hum Percept Perform. 44:626–644. 10.1037/xhp0000484. 29035072 PMC5897145

[ref18] Gaspelin N et al. 2023. The distractor positivity component and the inhibition of distracting stimuli. J Cogn Neurosci. 35:1693–1715. 10.1162/jocn_a_02051. 37677060

[ref19] Gerber EM, Golan T, Knight RT, Deouell LY. 2017. Cortical representation of persistent visual stimuli. NeuroImage. 161:67–79. 10.1016/j.neuroimage.2017.08.028. 28807872 PMC5957542

[ref20] Golan T et al. 2017. Increasing suppression of saccade-related transients along the human visual hierarchy. eLife. 6:e27819. 10.7554/eLife.27819. 28850030 PMC5576487

[ref21] Gondan M, Lange K, Rösler F, Röder B. 2004. The redundant target effect is affected by modality switch costs. Psychon Bull Rev. 11:307–313. 10.3758/BF03196575. 15260198

[ref22] Grill-Spector K, Henson R, Martin A. 2006. Repetition and the brain: neural models of stimulus-specific effects. Trends Cogn Sci. 10:14–23. 10.1016/j.tics.2005.11.006. 16321563

[ref23] Gutteling TP, Sillekens L, Lavie N, Jensen O. 2022. Alpha oscillations reflect suppression of distractors with increased perceptual load. Prog Neurobiol. 214:102285. 10.1016/j.pneurobio.2022.102285. 35533812 PMC7615060

[ref24] Hickey C, Theeuwes J. 2011. Context and competition in the capture of visual attention. Atten Percept Psychophys. 73:2053–2064. 10.3758/s13414-011-0168-9. 21739337 PMC3204046

[ref25] Hickey C, Di Lollo V, McDonald JJ. 2009. Electrophysiological indices of target and distractor processing in visual search. J Cogn Neurosci. 21:760–775. 10.1162/jocn.2009.21039. 18564048

[ref26] Hilimire MR, Mounts JRW, Parks NA, Corballis PM. 2011. Dynamics of target and distractor processing in visual search: evidence from event-related brain potentials. Neurosci Lett. 495:196–200. 10.1016/j.neulet.2011.03.064. 21457759

[ref27] Hopf J-M et al. 2000. Neural sources of focused attention in visual search. Cereb Cortex. 10:1233–1241. 10.1093/cercor/10.12.1233. 11073872

[ref28] Lee BB, Martin PR, Valberg A. 1988. The physiological basis of heterochromatic flicker photometry demonstrated in the ganglion cells of the macaque retina. J Physiol. 404:323–347. 3253435 10.1113/jphysiol.1988.sp017292 PMC1190828

[ref29] Leonard MK et al. 2024. Large-scale single-neuron speech sound encoding across the depth of human cortex. Nature. 626:593–602. 10.1038/s41586-023-06839-2. 38093008 PMC10866713

[ref30] Leszczyński M et al. 2020. Dissociation of broadband high-frequency activity and neuronal firing in the neocortex. Sci Adv. 6:eabb0977. 10.1126/sciadv.abb0977. 32851172 PMC7423365

[ref31] Liesefeld HR, Liesefeld AM, Töllner T, Müller HJ. 2017. Attentional capture in visual search: capture and post-capture dynamics revealed by EEG. NeuroImage. 156:166–173. 10.1016/j.neuroimage.2017.05.016. 28502842

[ref32] Liu B, Nobre AC, van Ede F. 2023. Microsaccades transiently lateralise EEG alpha activity. Prog Neurobiol. 224:102433. 10.1016/j.pneurobio.2023.102433. 36907349 PMC10074474

[ref33] Lowet E et al. 2018. Enhanced neural processing by covert attention only during microsaccades directed toward the attended stimulus. Neuron. 99:207–214.e3. 10.1016/j.neuron.2018.05.041. 29937279 PMC8415255

[ref34] Luck SJ, Hillyard SA. 1994. Spatial filtering during visual search: evidence from human electrophysiology. J Exp Psychol Hum Percept Perform. 20:1000–1014. 10.1037//0096-1523.20.5.1000. 7964526

[ref35] Luck SJ, Girelli M, McDermott MT, Ford MA. 1997. Bridging the gap between monkey neurophysiology and human perception: an ambiguity resolution theory of visual selective attention. Cognit Psychol. 33:64–87. 10.1006/cogp.1997.0660. 9212722

[ref36] Maniscalco B, Bang JW, Iravani L, Camps-Febrer F, Lau H. 2012. Does response interference depend on the subjective visibility of flanker distractors? Atten Percept Psychophys. 74:841–851. 10.3758/s13414-012-0291-2. 22477057

[ref37] Marturano F, Brigadoi S, Doro M, Dell’Acqua R, Sparacino G. 2020. A Time-Frequency Analysis for the Online Detection of the N2pc Event-Related Potential (ERP) Component in Individual EEG Datasets. In: 2020 42nd Annual International Conference of the IEEE Engineering in Medicine & Biology Society (EMBC). p. 1019–1022. [accessed 2023 Dec 14]. https://ieeexplore.ieee.org/abstract/document/9175462. 10.1109/EMBC44109.2020.9175462 33018158

[ref38] Mathôt S, Hickey C, Theeuwes J. 2010. From reorienting of attention to biased competition: evidence from hemifield effects. Atten Percept Psychophys. 72:651–657. 10.3758/APP.72.3.651. 20348571

[ref39] Miller KJ et al. 2014. Broadband changes in the cortical surface potential track activation of functionally diverse neuronal populations. NeuroImage. 85 Pt 2:711–720. 10.1016/j.neuroimage.2013.08.070. 24018305 PMC4347924

[ref42] Mounts JRW . 2000. Attentional capture by abrupt onsets and feature singletons produces inhibitory surrounds. Percept Psychophys. 62:1485–1493. 10.3758/BF03212148. 11143458

[ref43] Müller HJ, Heller D, Ziegler J. 1995. Visual search for singleton feature targets within and across feature dimensions. Percept Psychophys. 57:1–17. 10.3758/BF03211845. 7885801

[ref44] Olivers CNL, Humphreys GW. 2003. Visual marking inhibits singleton capture. Cognit Psychol. 47:1–42. 10.1016/S0010-0285(03)00003-3. 12852934

[ref45] Parra LC, Spence CD, Gerson AD, Sajda P. 2005. Recipes for the linear analysis of EEG. NeuroImage. 28:326–341. 10.1016/j.neuroimage.2005.05.032. 16084117

[ref46] Ray S, Niebur E, Hsiao SS, Sinai A, Crone NE. 2008. High-frequency gamma activity (80-150Hz) is increased in human cortex during selective attention. Clin Neurophysiol Off J Int Fed Clin Neurophysiol. 119:116–133. 10.1016/j.clinph.2007.09.136. PMC2444052 18037343

[ref47] Richter D, van Moorselaar D, Theeuwes J. 2024. Proactive distractor suppression in early visual cortex. eLife. 13:RP101733. 10.7554/eLife.101733.1. PMC11913444 40095805

[ref48] Rucci M, Iovin R, Poletti M, Santini F. 2007. Miniature eye movements enhance fine spatial detail. Nature. 447:852–855. 10.1038/nature05866. 17568745

[ref49] Sawaki R, Luck SJ. 2010. Capture versus suppression of attention by salient singletons: electrophysiological evidence for an automatic attend-to-me signal. Atten Percept Psychophys. 72:1455–1470. 10.3758/APP.72.6.1455. 20675793 PMC3705921

[ref50] Siegelman N, Bogaerts L, Kronenfeld O, Frost R. 2018. Redefining “learning” in statistical learning: what does an online measure reveal about the assimilation of visual regularities? Cogn Sci. 42:692–727. 10.1111/cogs.12556. 28986971 PMC5889756

[ref52] Szczepanski SM et al. 2014. Dynamic changes in phase-amplitude coupling facilitate spatial attention control in Fronto-parietal cortex. PLoS Biol. 12:e1001936. 10.1371/journal.pbio.1001936. 25157678 PMC4144794

[ref53] Tallon-Baudry C, Bertrand O, Hénaff M-A, Isnard J, Fischer C. 2005. Attention modulates gamma-band oscillations differently in the human lateral occipital cortex and fusiform gyrus. Cereb Cortex. 15:654–662. 10.1093/cercor/bhh167. 15371290

[ref40] van Moorselaar D, Slagter HA. 2020. Inhibition in selective attention. Ann N Y Acad Sci. 1464:204–221. 10.1111/nyas.14304. 31951294 PMC7155061

[ref41] van Moorselaar D, Lampers E, Cordesius E, Slagter HA. 2020. Neural mechanisms underlying expectation-dependent inhibition of distracting information. eLife. 9:e61048. 10.7554/eLife.61048. 33320084 PMC7758066

[ref51] van Moorselaar D, Slagter HA. 2019. Learning what is irrelevant or relevant: expectations facilitate distractor inhibition and target facilitation through distinct neural mechanisms. J Neurosci. 39:6953–6967. 10.1523/JNEUROSCI.0593-19.2019. 31270162 PMC6733564

[ref54] Vishne G, Gerber EM, Knight RT, Deouell LY. 2023. Distinct ventral stream and prefrontal cortex representational dynamics during sustained conscious visual perception. Cell Rep. 42:112752. 10.1016/j.celrep.2023.112752. 37422763 PMC10530642

[ref55] Wang B, Theeuwes J. 2018. How to inhibit a distractor location? Statistical learning versus active, top-down suppression. Atten Percept Psychophys. 80:860–870. 10.3758/s13414-018-1493-z. 29476331

[ref56] Watson DG, Humphreys GW. 2000. Visual marking: evidence for inhibition using a probe-dot detection paradigm. Percept Psychophys. 62:471–481. 10.3758/BF03212099. 10909238

[ref57] Wienke C et al. 2021. Mind-wandering is accompanied by both local sleep and enhanced processes of spatial attention allocation. Cereb Cortex Commun. 2:tgab001. 10.1093/texcom/tgab001. 34296151 PMC8153027

[ref58] Williams EJ . 1959. The comparison of regression variables. J R Stat Soc Ser B Stat Methodol. 21:396–399. 10.1111/j.2517-6161.1959.tb00346.x.

[ref59] Woodman GF, Arita JT, Luck SJ. 2009. A cuing study of the N2pc component: an index of attentional deployment to objects rather than spatial locations. Brain Res. 1297:101–111. 10.1016/j.brainres.2009.08.011. 19682440 PMC2758329

[ref60] Wöstmann M, Alavash M, Obleser J. 2019. Alpha oscillations in the human brain implement distractor suppression independent of target selection. J Neurosci. 39:9797–9805. 10.1523/JNEUROSCI.1954-19.2019. 31641052 PMC6891068

[ref61] Yu G, Herman JP, Katz LN, Krauzlis RJ. 2022. Microsaccades as a marker not a cause for attention-related modulation. eLife. 11:e74168. 10.7554/eLife.74168. 35289268 PMC8923660

[ref62] Yuval-Greenberg S, Tomer O, Keren AS, Nelken I, Deouell LY. 2008. Transient induced gamma-band response in EEG as a manifestation of miniature saccades. Neuron. 58:429–441. 10.1016/j.neuron.2008.03.027. 18466752

[ref63] Zhao C et al. 2023. Suppression of distracting inputs by visual-spatial cues is driven by anticipatory alpha activity. PLoS Biol. 21:e3002014. 10.1371/journal.pbio.3002014. 36888690 PMC10027229

